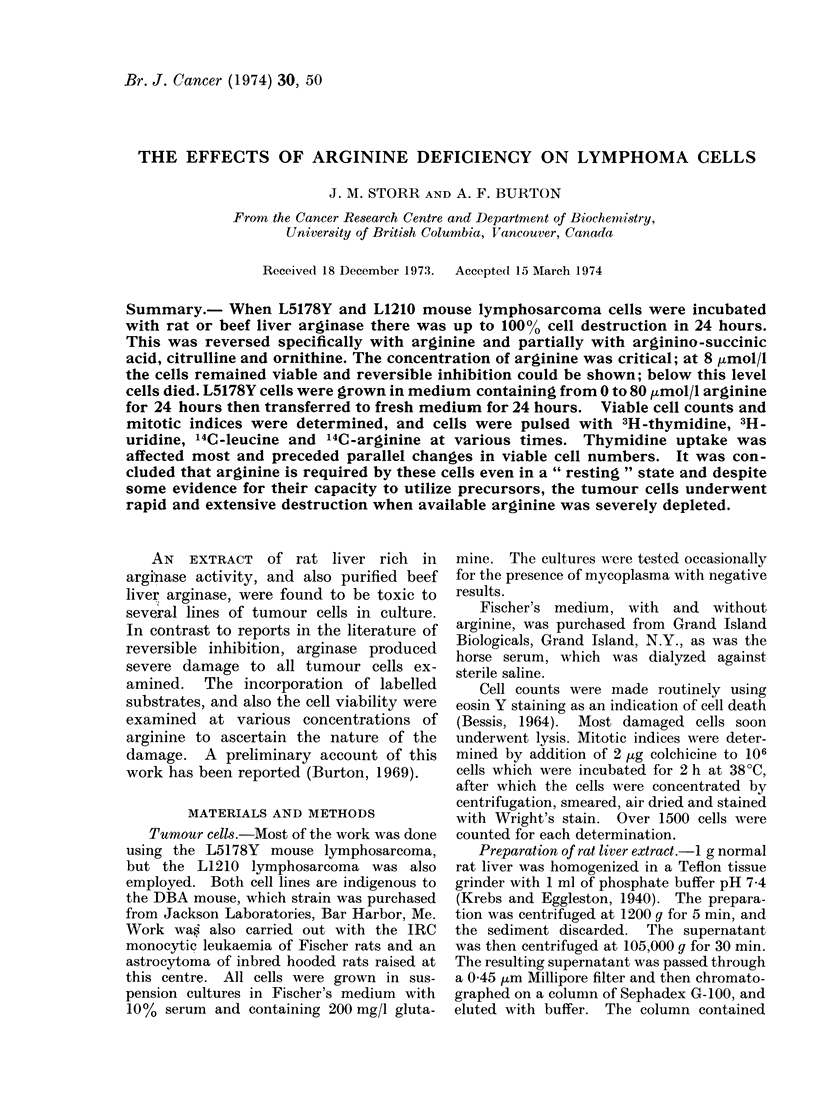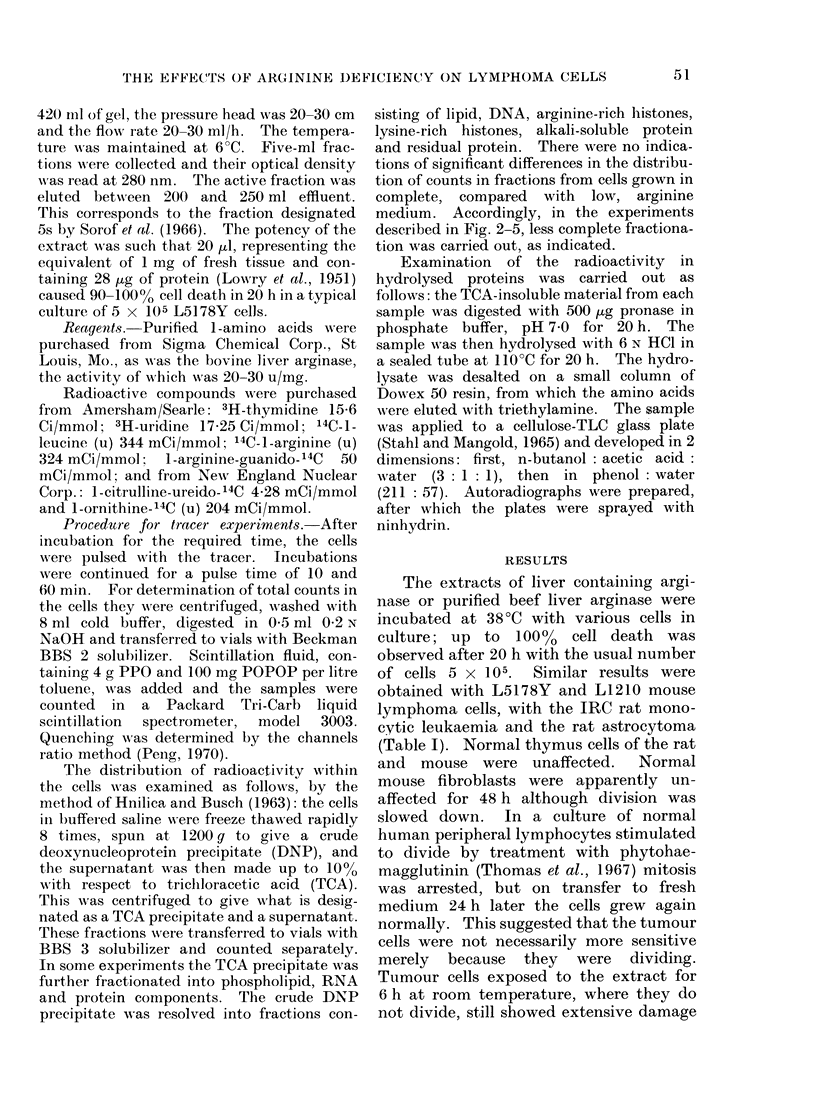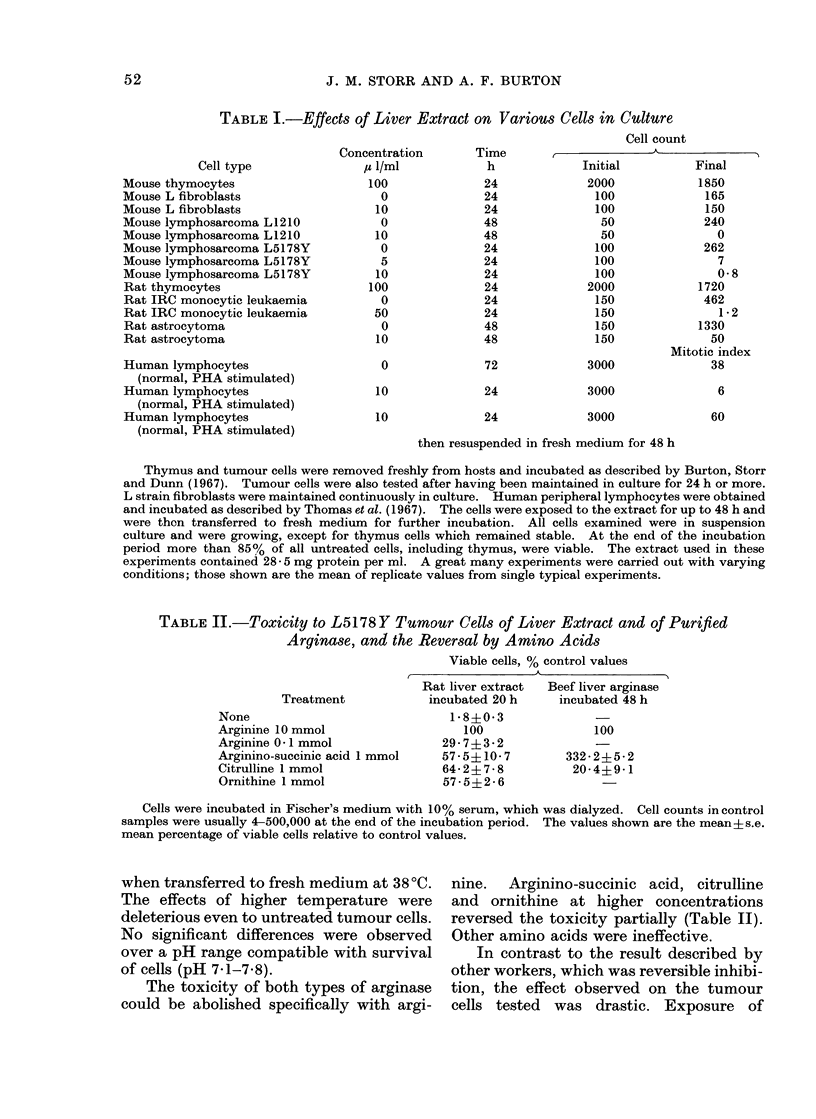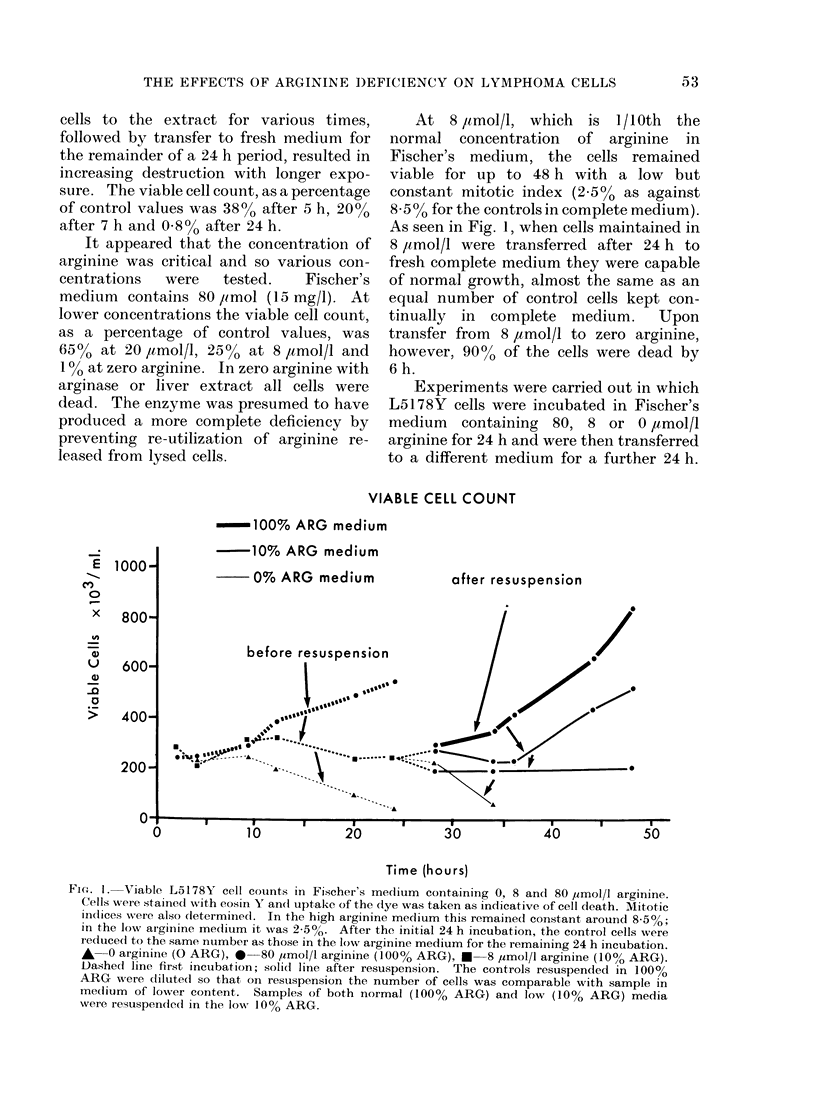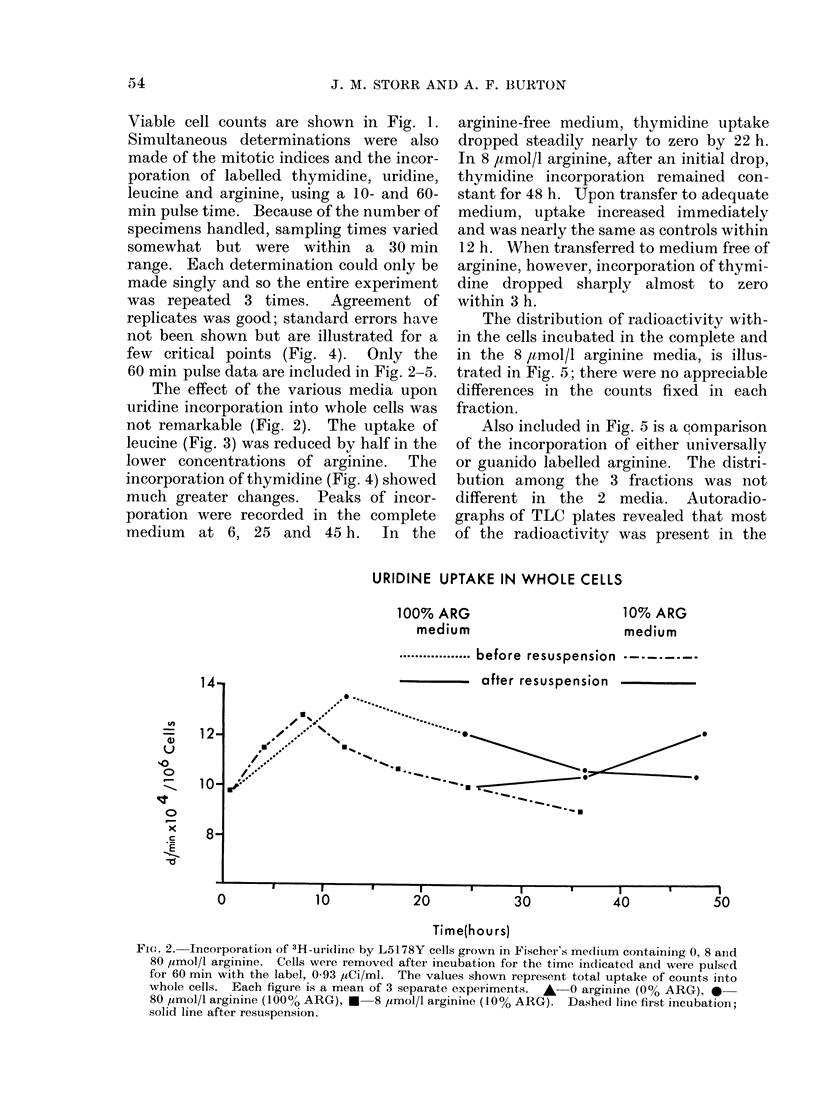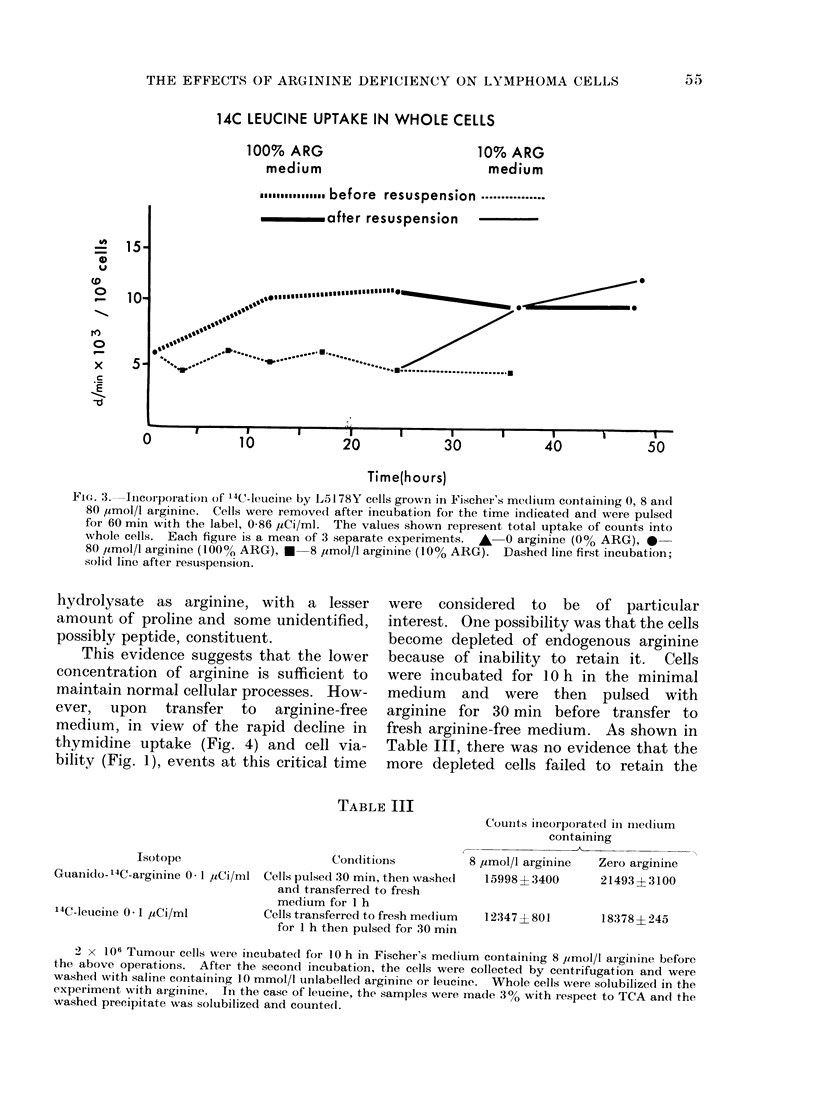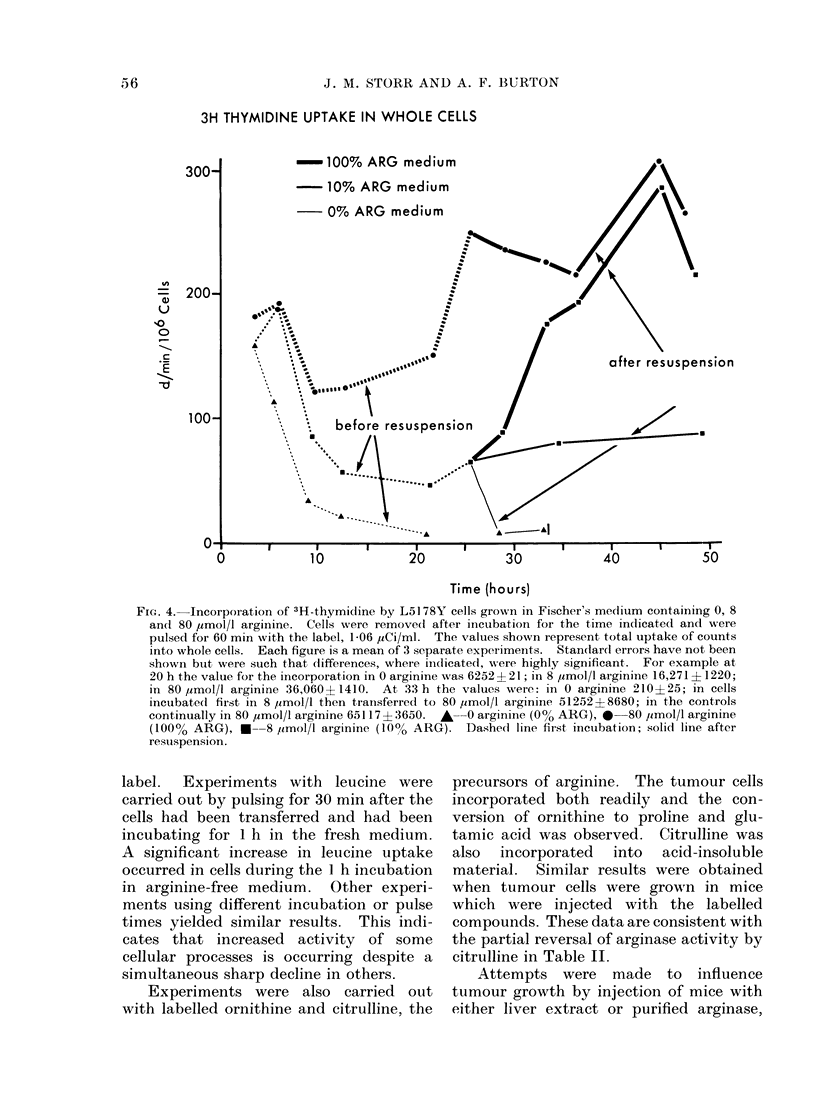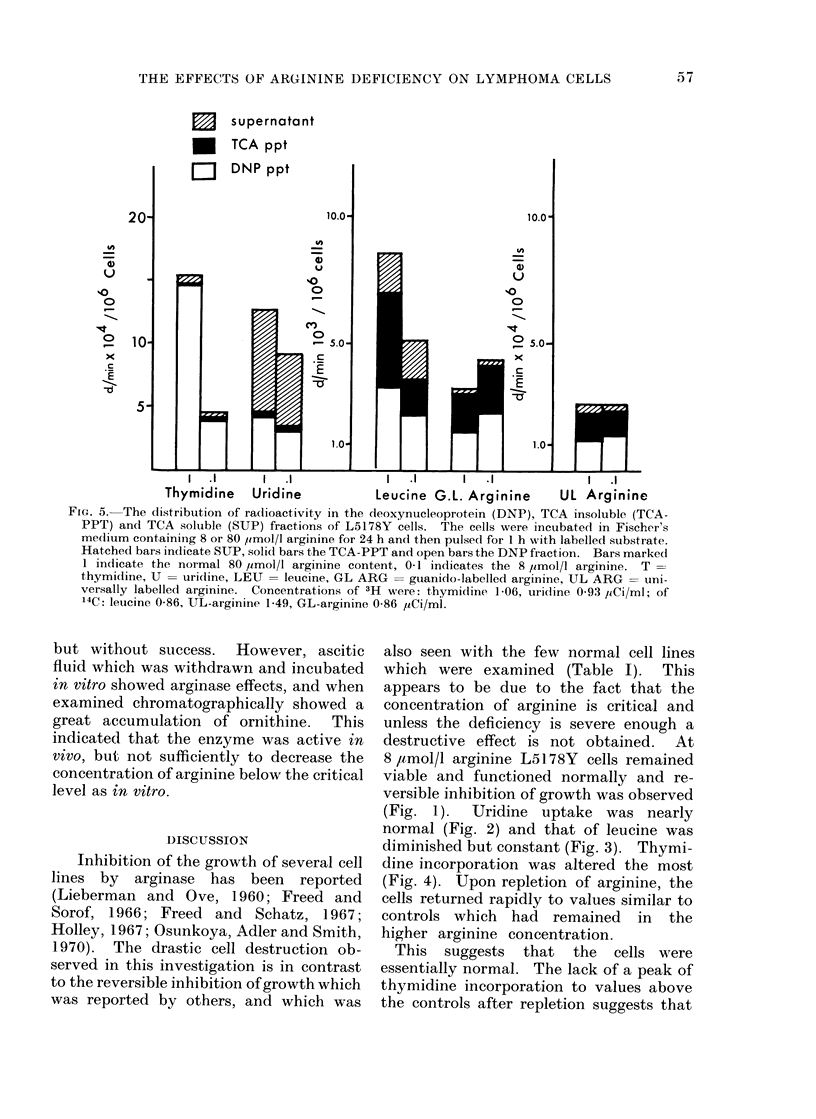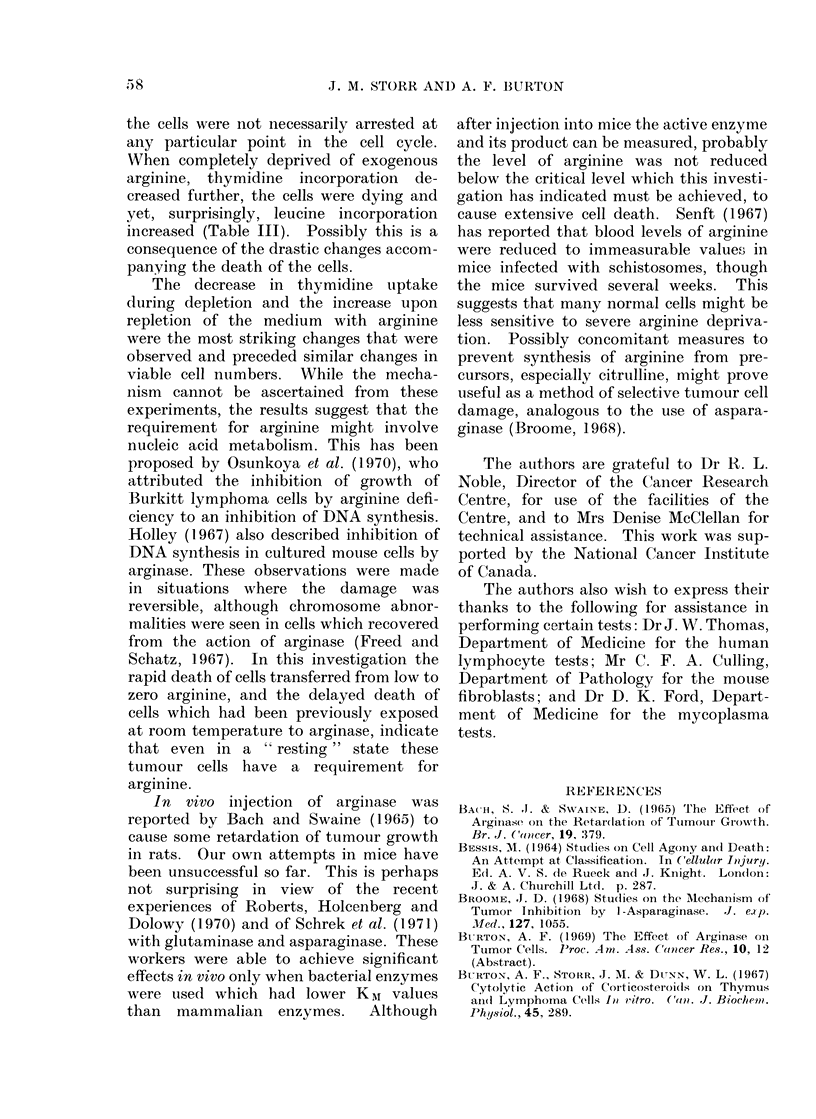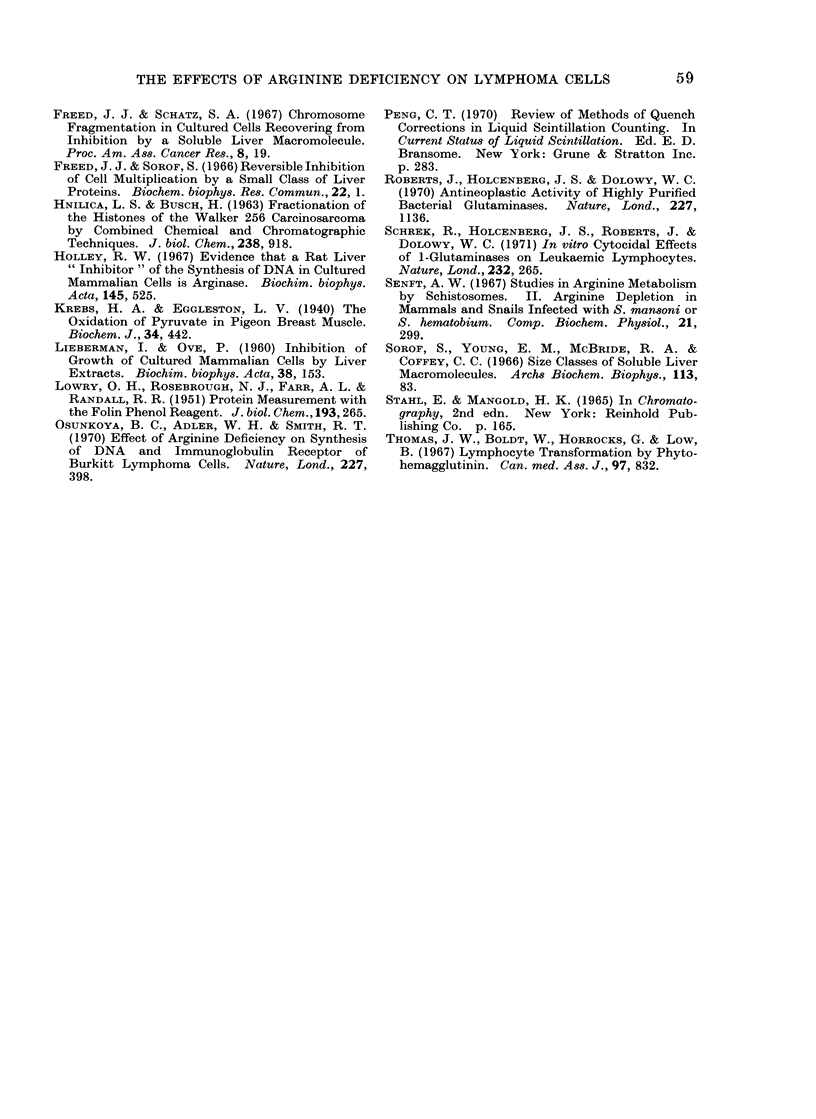# The Effects of Arginine Deficiency on Lymphoma Cells

**DOI:** 10.1038/bjc.1974.112

**Published:** 1974-07

**Authors:** J. M. Storr, A. F. Burton

## Abstract

When L5178Y and L1210 mouse lymphosarcoma cells were incubated with rat or beef liver arginase there was up to 100% cell destruction in 24 hours. This was reversed specifically with arginine and partially with arginino-succinic acid, citrulline and ornithine. The concentration of arginine was critical; at 8 μmol/l the cells remained viable and reversible inhibition could be shown; below this level cells died. L5178Y cells were grown in medium containing from 0 to 80 μmol/l arginine for 24 hours then transferred to fresh medium for 24 hours. Viable cell counts and mitotic indices were determined, and cells were pulsed with ^3^H-thymidine, ^3^H-uridine, ^14^C-leucine and ^14^C-arginine at various times. Thymidine uptake was affected most and preceded parallel changes in viable cell numbers. It was concluded that arginine is required by these cells even in a “resting” state and despite some evidence for their capacity to utilize precursors, the tumour cells underwent rapid and extensive destruction when available arginine was severely depleted.


*Br. J. Cancer* (1974) **30**, 50

### THE EFFECTS OF ARGININE DEFICIENCY ON LYMPHOMA CELLS

J. M. STORR AND A. F. BURTON

*From the Cancer Research Centre and Department of Biochemistry, University of British Columbia, Vancouver, Canada*

Received 18 December 1973. Accepted 15 March 1974

**Summary.— When L5178Y and L1210 mouse lymphosarcoma cells were incubated with rat or beef liver arginase there was up to 100% cell destruction in 24 hours. This was reversed specifically with arginine and partially with arginino-succinic acid, citrulline and ornithine. The concentration of arginine was critical; at 8 μmol/l the cells remained viable and reversible inhibition could be shown; below this level cells died. L5178Y cells were grown in medium containing from 0 to 80 μmol/l arginine for 24 hours then transferred to fresh medium for 24 hours. Viable cell counts and mitotic indices were determined, and cells were pulsed with $^{3}$H-thymidine, $^{3}$H-uridine, $^{14}$C-leucine and $^{14}$C-arginine at various times. Thymidine uptake was affected most and preceded parallel changes in viable cell numbers. It was concluded that arginine is required by these cells even in a " resting " state and despite some evidence for their capacity to utilize precursors, the tumour cells underwent rapid and extensive destruction when available arginine was severely depleted.**

AN EXTRACT of rat liver rich in arginase activity, and also purified beef liver arginase, were found to be toxic to several lines of tumour cells in culture. In contrast to reports in the literature of reversible inhibition, arginase produced severe damage to all tumour cells examined. The incorporation of labelled substrates, and also the cell viability were examined at various concentrations of arginine to ascertain the nature of the damage. A preliminary account of this work has been reported (Burton, 1969).

### MATERIALS AND METHODS

*Tumour cells.*—Most of the work was done using the L5178Y mouse lymphosarcoma, but the L1210 lymphosarcoma was also employed. Both cell lines are indigenous to the DBA mouse, which strain was purchased from Jackson Laboratories, Bar Harbor, Me. Work was also carried out with the IRC monocytic leukaemia of Fischer rats and an astrocytoma of inbred hooded rats raised at this centre. All cells were grown in suspension cultures in Fischer's medium with 10% serum and containing 200 mg/l glutamine. The cultures were tested occasionally for the presence of mycoplasma with negative results.

Fischer's medium, with and without arginine, was purchased from Grand Island Biologicals, Grand Island, N.Y., as was the horse serum, which was dialyzed against sterile saline.

Cell counts were made routinely using eosin Y staining as an indication of cell death (Bessis, 1964). Most damaged cells soon underwent lysis. Mitotic indices were determined by addition of 2 μg colchicine to $10^{6}$ cells which were incubated for 2 h at 38°C, after which the cells were concentrated by centrifugation, smeared, air dried and stained with Wright's stain. Over 1500 cells were counted for each determination.

*Preparation of rat liver extract.*—1 g normal rat liver was homogenized in a Teflon tissue grinder with 1 ml of phosphate buffer pH 7·4 (Krebs and Eggleston, 1940). The preparation was centrifuged at 1200 *g* for 5 min, and the sediment discarded. The supernatant was then centrifuged at 105,000 *g* for 30 min. The resulting supernatant was passed through a 0·45 μm Millipore filter and then chromatographed on a column of Sephadex G-100, and eluted with buffer. The column contained

420 ml of gel, the pressure head was 20–30 cm and the flow rate 20–30 ml/h. The temperature was maintained at 6°C. Five-ml fractions were collected and their optical density was read at 280 nm. The active fraction was eluted between 200 and 250 ml effluent. This corresponds to the fraction designated 5s by Sorof *et al.* (1966). The potency of the extract was such that 20 $\mu$l, representing the equivalent of 1 mg of fresh tissue and containing 28 $\mu$g of protein (Lowry *et al.*, 1951) caused 90–100% cell death in 20 h in a typical culture of $5 \times 10^5$ L5178Y cells.

*Reagents.*—Purified l-amino acids were purchased from Sigma Chemical Corp., St Louis, Mo., as was the bovine liver arginase, the activity of which was 20–30 u/mg.

Radioactive compounds were purchased from Amersham/Searle: $^3$H-thymidine 15·6 Ci/mmol; $^3$H-uridine 17·25 Ci/mmol; $^{14}$C-l-leucine (u) 344 mCi/mmol; $^{14}$C-l-arginine (u) 324 mCi/mmol; l-arginine-guanido-$^{14}$C 50 mCi/mmol; and from New England Nuclear Corp.: l-citrulline-ureido-$^{14}$C 4·28 mCi/mmol and l-ornithine-$^{14}$C (u) 204 mCi/mmol.

*Procedure for tracer experiments.*—After incubation for the required time, the cells were pulsed with the tracer. Incubations were continued for a pulse time of 10 and 60 min. For determination of total counts in the cells they were centrifuged, washed with 8 ml cold buffer, digested in 0·5 ml 0·2 N NaOH and transferred to vials with Beckman BBS 2 solubilizer. Scintillation fluid, containing 4 g PPO and 100 mg POPOP per litre toluene, was added and the samples were counted in a Packard Tri-Carb liquid scintillation spectrometer, model 3003. Quenching was determined by the channels ratio method (Peng, 1970).

The distribution of radioactivity within the cells was examined as follows, by the method of Hnilica and Busch (1963): the cells in buffered saline were freeze thawed rapidly 8 times, spun at 1200 *g* to give a crude deoxynucleoprotein precipitate (DNP), and the supernatant was then made up to 10% with respect to trichloracetic acid (TCA). This was centrifuged to give what is designated as a TCA precipitate and a supernatant. These fractions were transferred to vials with BBS 3 solubilizer and counted separately. In some experiments the TCA precipitate was further fractionated into phospholipid, RNA and protein components. The crude DNP precipitate was resolved into fractions consisting of lipid, DNA, arginine-rich histones, lysine-rich histones, alkali-soluble protein and residual protein. There were no indications of significant differences in the distribution of counts in fractions from cells grown in complete, compared with low, arginine medium. Accordingly, in the experiments described in Fig. 2–5, less complete fractionation was carried out, as indicated.

Examination of the radioactivity in hydrolysed proteins was carried out as follows: the TCA-insoluble material from each sample was digested with 500 $\mu$g pronase in phosphate buffer, pH 7·0 for 20 h. The sample was then hydrolysed with 6 N HCl in a sealed tube at 110°C for 20 h. The hydrolysate was desalted on a small column of Dowex 50 resin, from which the amino acids were eluted with triethylamine. The sample was applied to a cellulose-TLC glass plate (Stahl and Mangold, 1965) and developed in 2 dimensions: first, n-butanol : acetic acid : water (3 : 1 : 1), then in phenol : water (211 : 57). Autoradiographs were prepared, after which the plates were sprayed with ninhydrin.

### RESULTS

The extracts of liver containing arginase or purified beef liver arginase were incubated at 38°C with various cells in culture; up to 100% cell death was observed after 20 h with the usual number of cells $5 \times 10^5$. Similar results were obtained with L5178Y and L1210 mouse lymphoma cells, with the IRC rat monocytic leukaemia and the rat astrocytoma (Table I). Normal thymus cells of the rat and mouse were unaffected. Normal mouse fibroblasts were apparently unaffected for 48 h although division was slowed down. In a culture of normal human peripheral lymphocytes stimulated to divide by treatment with phytohaemagglutinin (Thomas *et al.*, 1967) mitosis was arrested, but on transfer to fresh medium 24 h later the cells grew again normally. This suggested that the tumour cells were not necessarily more sensitive merely because they were dividing. Tumour cells exposed to the extract for 6 h at room temperature, where they do not divide, still showed extensive damage

TABLE I.—*Effects of Liver Extract on Various Cells in Culture*

| Cell type | Concentration µ l/ml | Time h | Cell count Initial | Final |
|---|---|---|---|---|
| Mouse thymocytes | 100 | 24 | 2000 | 1850 |
| Mouse L fibroblasts | 0 | 24 | 100 | 165 |
| Mouse L fibroblasts | 10 | 24 | 100 | 150 |
| Mouse lymphosarcoma L1210 | 0 | 48 | 50 | 240 |
| Mouse lymphosarcoma L1210 | 10 | 48 | 50 | 0 |
| Mouse lymphosarcoma L5178Y | 0 | 24 | 100 | 262 |
| Mouse lymphosarcoma L5178Y | 5 | 24 | 100 | 7 |
| Mouse lymphosarcoma L5178Y | 10 | 24 | 100 | 0·8 |
| Rat thymocytes | 100 | 24 | 2000 | 1720 |
| Rat IRC monocytic leukaemia | 0 | 24 | 150 | 462 |
| Rat IRC monocytic leukaemia | 50 | 24 | 150 | 1·2 |
| Rat astrocytoma | 0 | 48 | 150 | 1330 |
| Rat astrocytoma | 10 | 48 | 150 | 50 |
| | | | | Mitotic index |
| Human lymphocytes (normal, PHA stimulated) | 0 | 72 | 3000 | 38 |
| Human lymphocytes (normal, PHA stimulated) | 10 | 24 | 3000 | 6 |
| Human lymphocytes (normal, PHA stimulated) | 10 | 24 then resuspended in fresh medium for 48 h | 3000 | 60 |

Thymus and tumour cells were removed freshly from hosts and incubated as described by Burton, Storr and Dunn (1967). Tumour cells were also tested after having been maintained in culture for 24 h or more. L strain fibroblasts were maintained continuously in culture. Human peripheral lymphocytes were obtained and incubated as described by Thomas *et al.* (1967). The cells were exposed to the extract for up to 48 h and were then transferred to fresh medium for further incubation. All cells examined were in suspension culture and were growing, except for thymus cells which remained stable. At the end of the incubation period more than 85% of all untreated cells, including thymus, were viable. The extract used in these experiments contained 28·5 mg protein per ml. A great many experiments were carried out with varying conditions; those shown are the mean of replicate values from single typical experiments.

TABLE II.—*Toxicity to L5178Y Tumour Cells of Liver Extract and of Purified Arginase, and the Reversal by Amino Acids*

| Treatment | Viable cells, % control values: Rat liver extract incubated 20 h | Beef liver arginase incubated 48 h |
|---|---|---|
| None | 1·8±0·3 | — |
| Arginine 10 mmol | 100 | 100 |
| Arginine 0·1 mmol | 29·7±3·2 | — |
| Arginino-succinic acid 1 mmol | 57·5±10·7 | 332·2±5·2 |
| Citrulline 1 mmol | 64·2±7·8 | 20·4±9·1 |
| Ornithine 1 mmol | 57·5±2·6 | — |

Cells were incubated in Fischer's medium with 10% serum, which was dialyzed. Cell counts in control samples were usually 4–500,000 at the end of the incubation period. The values shown are the mean±s.e. mean percentage of viable cells relative to control values.

when transferred to fresh medium at 38 °C. The effects of higher temperature were deleterious even to untreated tumour cells. No significant differences were observed over a pH range compatible with survival of cells (pH 7·1–7·8).

The toxicity of both types of arginase could be abolished specifically with arginine. Arginino-succinic acid, citrulline and ornithine at higher concentrations reversed the toxicity partially (Table II). Other amino acids were ineffective.

In contrast to the result described by other workers, which was reversible inhibition, the effect observed on the tumour cells tested was drastic. Exposure of cells to the extract for various times, followed by transfer to fresh medium for the remainder of a 24 h period, resulted in increasing destruction with longer exposure. The viable cell count, as a percentage of control values was 38% after 5 h, 20% after 7 h and 0·8% after 24 h.

It appeared that the concentration of arginine was critical and so various concentrations were tested. Fischer's medium contains 80 μmol (15 mg/l). At lower concentrations the viable cell count, as a percentage of control values, was 65% at 20 μmol/l, 25% at 8 μmol/l and 1% at zero arginine. In zero arginine with arginase or liver extract all cells were dead. The enzyme was presumed to have produced a more complete deficiency by preventing re-utilization of arginine released from lysed cells.

At 8 μmol/l, which is 1/10th the normal concentration of arginine in Fischer's medium, the cells remained viable for up to 48 h with a low but constant mitotic index (2·5% as against 8·5% for the controls in complete medium). As seen in Fig. 1, when cells maintained in 8 μmol/l were transferred after 24 h to fresh complete medium they were capable of normal growth, almost the same as an equal number of control cells kept continually in complete medium. Upon transfer from 8 μmol/l to zero arginine, however, 90% of the cells were dead by 6 h.

Experiments were carried out in which L5178Y cells were incubated in Fischer's medium containing 80, 8 or 0 μmol/l arginine for 24 h and were then transferred to a different medium for a further 24 h.

FIG. 1.—Viable L5178Y cell counts in Fischer's medium containing 0, 8 and 80 μmol/l arginine. Cells were stained with eosin Y and uptake of the dye was taken as indicative of cell death. Mitotic indices were also determined. In the high arginine medium this remained constant around 8·5%; in the low arginine medium it was 2·5%. After the initial 24 h incubation, the control cells were reduced to the same number as those in the low arginine medium for the remaining 24 h incubation. ▲—0 arginine (0 ARG), ●—80 μmol/l arginine (100% ARG), ■—8 μmol/l arginine (10% ARG). Dashed line first incubation; solid line after resuspension. The controls resuspended in 100% ARG were diluted so that on resuspension the number of cells was comparable with sample in medium of lower content. Samples of both normal (100% ARG) and low (10% ARG) media were resuspended in the low 10% ARG.

Viable cell counts are shown in Fig. 1. Simultaneous determinations were also made of the mitotic indices and the incorporation of labelled thymidine, uridine, leucine and arginine, using a 10- and 60-min pulse time. Because of the number of specimens handled, sampling times varied somewhat but were within a 30 min range. Each determination could only be made singly and so the entire experiment was repeated 3 times. Agreement of replicates was good; standard errors have not been shown but are illustrated for a few critical points (Fig. 4). Only the 60 min pulse data are included in Fig. 2–5.

The effect of the various media upon uridine incorporation into whole cells was not remarkable (Fig. 2). The uptake of leucine (Fig. 3) was reduced by half in the lower concentrations of arginine. The incorporation of thymidine (Fig. 4) showed much greater changes. Peaks of incorporation were recorded in the complete medium at 6, 25 and 45 h. In the arginine-free medium, thymidine uptake dropped steadily nearly to zero by 22 h. In 8 μmol/l arginine, after an initial drop, thymidine incorporation remained constant for 48 h. Upon transfer to adequate medium, uptake increased immediately and was nearly the same as controls within 12 h. When transferred to medium free of arginine, however, incorporation of thymidine dropped sharply almost to zero within 3 h.

The distribution of radioactivity within the cells incubated in the complete and in the 8 μmol/l arginine media, is illustrated in Fig. 5; there were no appreciable differences in the counts fixed in each fraction.

Also included in Fig. 5 is a comparison of the incorporation of either universally or guanido labelled arginine. The distribution among the 3 fractions was not different in the 2 media. Autoradiographs of TLC plates revealed that most of the radioactivity was present in the

FIG. 2.—Incorporation of $^3$H-uridine by L5178Y cells grown in Fischer's medium containing 0, 8 and 80 μmol/l arginine. Cells were removed after incubation for the time indicated and were pulsed for 60 min with the label, 0·93 μCi/ml. The values shown represent total uptake of counts into whole cells. Each figure is a mean of 3 separate experiments. ▲—0 arginine (0% ARG), ●—80 μmol/l arginine (100% ARG), ■—8 μmol/l arginine (10% ARG). Dashed line first incubation; solid line after resuspension.

FIG. 3.—Incorporation of $^{14}$C-leucine by L5178Y cells grown in Fischer's medium containing 0, 8 and 80 $\mu$mol/l arginine. Cells were removed after incubation for the time indicated and were pulsed for 60 min with the label, 0·86 $\mu$Ci/ml. The values shown represent total uptake of counts into whole cells. Each figure is a mean of 3 separate experiments. ▲—0 arginine (0% ARG), ●—80 $\mu$mol/l arginine (100% ARG), ■—8 $\mu$mol/l arginine (10% ARG). Dashed line first incubation; solid line after resuspension.

hydrolysate as arginine, with a lesser amount of proline and some unidentified, possibly peptide, constituent.

This evidence suggests that the lower concentration of arginine is sufficient to maintain normal cellular processes. However, upon transfer to arginine-free medium, in view of the rapid decline in thymidine uptake (Fig. 4) and cell viability (Fig. 1), events at this critical time were considered to be of particular interest. One possibility was that the cells become depleted of endogenous arginine because of inability to retain it. Cells were incubated for 10 h in the minimal medium and were then pulsed with arginine for 30 min before transfer to fresh arginine-free medium. As shown in Table III, there was no evidence that the more depleted cells failed to retain the

TABLE III

| Isotope | Conditions | Counts incorporated in medium containing | |
|---|---|---|---|
| | | 8 $\mu$mol/l arginine | Zero arginine |
| Guanido-$^{14}$C-arginine 0·1 $\mu$Ci/ml | Cells pulsed 30 min, then washed and transferred to fresh medium for 1 h | 15998±3400 | 21493±3100 |
| $^{14}$C-leucine 0·1 $\mu$Ci/ml | Cells transferred to fresh medium for 1 h then pulsed for 30 min | 12347±801 | 18378±245 |

2 × 10$^6$ Tumour cells were incubated for 10 h in Fischer's medium containing 8 $\mu$mol/l arginine before the above operations. After the second incubation, the cells were collected by centrifugation and were washed with saline containing 10 mmol/l unlabelled arginine or leucine. Whole cells were solubilized in the experiment with arginine. In the case of leucine, the samples were made 3% with respect to TCA and the washed precipitate was solubilized and counted.

FIG. 4.—Incorporation of $^3$H-thymidine by L5178Y cells grown in Fischer's medium containing 0, 8 and 80 $\mu$mol/l arginine. Cells were removed after incubation for the time indicated and were pulsed for 60 min with the label, 1·06 $\mu$Ci/ml. The values shown represent total uptake of counts into whole cells. Each figure is a mean of 3 separate experiments. Standard errors have not been shown but were such that differences, where indicated, were highly significant. For example at 20 h the value for the incorporation in 0 arginine was 6252$\pm$21; in 8 $\mu$mol/l arginine 16,271$\pm$1220; in 80 $\mu$mol/l arginine 36,060$\pm$1410. At 33 h the values were: in 0 arginine 210$\pm$25; in cells incubated first in 8 $\mu$mol/l then transferred to 80 $\mu$mol/l arginine 51252$\pm$8680; in the controls continually in 80 $\mu$mol/l arginine 65117$\pm$3650. ▲—0 arginine (0% ARG), ●—80 $\mu$mol/l arginine (100% ARG), ■—8 $\mu$mol/l arginine (10% ARG). Dashed line first incubation; solid line after resuspension.

label. Experiments with leucine were carried out by pulsing for 30 min after the cells had been transferred and had been incubating for 1 h in the fresh medium. A significant increase in leucine uptake occurred in cells during the 1 h incubation in arginine-free medium. Other experiments using different incubation or pulse times yielded similar results. This indicates that increased activity of some cellular processes is occurring despite a simultaneous sharp decline in others.

Experiments were also carried out with labelled ornithine and citrulline, the precursors of arginine. The tumour cells incorporated both readily and the conversion of ornithine to proline and glutamic acid was observed. Citrulline was also incorporated into acid-insoluble material. Similar results were obtained when tumour cells were grown in mice which were injected with the labelled compounds. These data are consistent with the partial reversal of arginase activity by citrulline in Table II.

Attempts were made to influence tumour growth by injection of mice with either liver extract or purified arginase,

FIG. 5.—The distribution of radioactivity in the deoxynucleoprotein (DNP), TCA insoluble (TCA-PPT) and TCA soluble (SUP) fractions of L5178Y cells. The cells were incubated in Fischer's medium containing 8 or 80 μmol/l arginine for 24 h and then pulsed for 1 h with labelled substrate. Hatched bars indicate SUP, solid bars the TCA-PPT and open bars the DNP fraction. Bars marked 1 indicate the normal 80 μmol/l arginine content, 0·1 indicates the 8 μmol/l arginine. T = thymidine, U = uridine, LEU = leucine, GL ARG = guanido-labelled arginine, UL ARG = universally labelled arginine. Concentrations of $^{3}H$ were: thymidine 1·06, uridine 0·93 μCi/ml; of $^{14}C$: leucine 0·86, UL-arginine 1·49, GL-arginine 0·86 μCi/ml.

but without success. However, ascitic fluid which was withdrawn and incubated *in vitro* showed arginase effects, and when examined chromatographically showed a great accumulation of ornithine. This indicated that the enzyme was active *in vivo*, but not sufficiently to decrease the concentration of arginine below the critical level as *in vitro*.

### DISCUSSION

Inhibition of the growth of several cell lines by arginase has been reported (Lieberman and Ove, 1960; Freed and Sorof, 1966; Freed and Schatz, 1967; Holley, 1967; Osunkoya, Adler and Smith, 1970). The drastic cell destruction observed in this investigation is in contrast to the reversible inhibition of growth which was reported by others, and which was also seen with the few normal cell lines which were examined (Table I). This appears to be due to the fact that the concentration of arginine is critical and unless the deficiency is severe enough a destructive effect is not obtained. At 8 μmol/l arginine L5178Y cells remained viable and functioned normally and reversible inhibition of growth was observed (Fig. 1). Uridine uptake was nearly normal (Fig. 2) and that of leucine was diminished but constant (Fig. 3). Thymidine incorporation was altered the most (Fig. 4). Upon repletion of arginine, the cells returned rapidly to values similar to controls which had remained in the higher arginine concentration.

This suggests that the cells were essentially normal. The lack of a peak of thymidine incorporation to values above the controls after repletion suggests that the cells were not necessarily arrested at any particular point in the cell cycle. When completely deprived of exogenous arginine, thymidine incorporation decreased further, the cells were dying and yet, surprisingly, leucine incorporation increased (Table III). Possibly this is a consequence of the drastic changes accompanying the death of the cells.

The decrease in thymidine uptake during depletion and the increase upon repletion of the medium with arginine were the most striking changes that were observed and preceded similar changes in viable cell numbers. While the mechanism cannot be ascertained from these experiments, the results suggest that the requirement for arginine might involve nucleic acid metabolism. This has been proposed by Osunkoya *et al.* (1970), who attributed the inhibition of growth of Burkitt lymphoma cells by arginine deficiency to an inhibition of DNA synthesis. Holley (1967) also described inhibition of DNA synthesis in cultured mouse cells by arginase. These observations were made in situations where the damage was reversible, although chromosome abnormalities were seen in cells which recovered from the action of arginase (Freed and Schatz, 1967). In this investigation the rapid death of cells transferred from low to zero arginine, and the delayed death of cells which had been previously exposed at room temperature to arginase, indicate that even in a " resting " state these tumour cells have a requirement for arginine.

*In vivo* injection of arginase was reported by Bach and Swaine (1965) to cause some retardation of tumour growth in rats. Our own attempts in mice have been unsuccessful so far. This is perhaps not surprising in view of the recent experiences of Roberts, Holcenberg and Dolowy (1970) and of Schrek *et al.* (1971) with glutaminase and asparaginase. These workers were able to achieve significant effects *in vivo* only when bacterial enzymes were used which had lower $K_M$ values than mammalian enzymes. Although after injection into mice the active enzyme and its product can be measured, probably the level of arginine was not reduced below the critical level which this investigation has indicated must be achieved, to cause extensive cell death. Senft (1967) has reported that blood levels of arginine were reduced to immeasurable values in mice infected with schistosomes, though the mice survived several weeks. This suggests that many normal cells might be less sensitive to severe arginine deprivation. Possibly concomitant measures to prevent synthesis of arginine from precursors, especially citrulline, might prove useful as a method of selective tumour cell damage, analogous to the use of asparaginase (Broome, 1968).

The authors are grateful to Dr R. L. Noble, Director of the Cancer Research Centre, for use of the facilities of the Centre, and to Mrs Denise McClellan for technical assistance. This work was supported by the National Cancer Institute of Canada.

The authors also wish to express their thanks to the following for assistance in performing certain tests: Dr J. W. Thomas, Department of Medicine for the human lymphocyte tests; Mr C. F. A. Culling, Department of Pathology for the mouse fibroblasts; and Dr D. K. Ford, Department of Medicine for the mycoplasma tests.

### REFERENCES

BACH, S. J. & SWAINE, D. (1965) The Effect of Arginase on the Retardation of Tumour Growth. *Br. J. Cancer*, **19**, 379.

BESSIS, M. (1964) Studies on Cell Agony and Death: An Attempt at Classification. In *Cellular Injury*. Ed. A. V. S. de Rueck and J. Knight. London: J. & A. Churchill Ltd. p. 287.

BROOME, J. D. (1968) Studies on the Mechanism of Tumor Inhibition by l-Asparaginase. *J. exp. Med.*, **127**, 1055.

BURTON, A. F. (1969) The Effect of Arginase on Tumor Cells. *Proc. Am. Ass. Cancer Res.*, **10**, 12 (Abstract).

BURTON, A. F., STORR, J. M. & DUNN, W. L. (1967) Cytolytic Action of Corticosteroids on Thymus and Lymphoma Cells *In vitro*. *Can. J. Biochem. Physiol.*, **45**, 289.

FREED, J. J. & SCHATZ, S. A. (1967) Chromosome Fragmentation in Cultured Cells Recovering from Inhibition by a Soluble Liver Macromolecule. *Proc. Am. Ass. Cancer Res.*, **8**, 19.

FREED, J. J. & SOROF, S. (1966) Reversible Inhibition of Cell Multiplication by a Small Class of Liver Proteins. *Biochem. biophys. Res. Commun.*, **22**, 1.

HNILICA, L. S. & BUSCH, H. (1963) Fractionation of the Histones of the Walker 256 Carcinosarcoma by Combined Chemical and Chromatographic Techniques. *J. biol. Chem.*, **238**, 918.

HOLLEY, R. W. (1967) Evidence that a Rat Liver " Inhibitor " of the Synthesis of DNA in Cultured Mammalian Cells is Arginase. *Biochim. biophys. Acta*, **145**, 525.

KREBS, H. A. & EGGLESTON, L. V. (1940) The Oxidation of Pyruvate in Pigeon Breast Muscle. *Biochem. J.*, **34**, 442.

LIEBERMAN, I. & OVE, P. (1960) Inhibition of Growth of Cultured Mammalian Cells by Liver Extracts. *Biochim. biophys. Acta*, **38**, 153.

LOWRY, O. H., ROSEBROUGH, N. J., FARR, A. L. & RANDALL, R. R. (1951) Protein Measurement with the Folin Phenol Reagent. *J. biol. Chem.*, **193**, 265.

OSUNKOYA, B. C., ADLER, W. H. & SMITH, R. T. (1970) Effect of Arginine Deficiency on Synthesis of DNA and Immunoglobulin Receptor of Burkitt Lymphoma Cells. *Nature, Lond.*, **227**, 398.

PENG, C. T. (1970) Review of Methods of Quench Corrections in Liquid Scintillation Counting. In *Current Status of Liquid Scintillation.* Ed. E. D. Bransome. New York: Grune & Stratton Inc. p. 283.

ROBERTS, J., HOLCENBERG, J. S. & DOLOWY, W. C. (1970) Antineoplastic Activity of Highly Purified Bacterial Glutaminases. *Nature, Lond.*, **227**, 1136.

SCHREK, R., HOLCENBERG, J. S., ROBERTS, J. & DOLOWY, W. C. (1971) *In vitro* Cytocidal Effects of 1-Glutaminases on Leukaemic Lymphocytes. *Nature, Lond.*, **232**, 265.

SENFT, A. W. (1967) Studies in Arginine Metabolism by Schistosomes. II. Arginine Depletion in Mammals and Snails Infected with *S. mansoni* or *S. hematobium. Comp. Biochem. Physiol.*, **21**, 299.

SOROF, S., YOUNG, E. M., MCBRIDE, R. A. & COFFEY, C. C. (1966) Size Classes of Soluble Liver Macromolecules. *Archs Biochem. Biophys.*, **113**, 83.

STAHL, E. & MANGOLD, H. K. (1965) In *Chromatography*, 2nd edn. New York: Reinhold Publishing Co. p. 165.

THOMAS, J. W., BOLDT, W., HORROCKS, G. & LOW, B. (1967) Lymphocyte Transformation by Phytohemagglutinin. *Can. med. Ass. J.*, **97**, 832.